# Effects of a supplement designed to increase ATP levels on muscle strength, power output, and endurance

**DOI:** 10.1186/1550-2783-5-3

**Published:** 2008-01-29

**Authors:** Trent J Herda, Eric D Ryan, Jeffrey R Stout, Joel T Cramer

**Affiliations:** 1 Department of Health and Exercise Science, University of Oklahoma, Norman, USA

## Abstract

**Background:**

The present study examined the acute effects of a nutritional supplement intended to improve adenosine triphosphate (ATP) concentrations on vertical jump height, isometric strength of the leg extensors, leg extension endurance, and forearm flexion endurance.

**Methods:**

Twenty-four healthy men (mean age ± SD = 23 ± 4 yrs, stature = 181 ± 7 cm, and body mass = 82 ± 12 kg) volunteered to complete a familiarization trial plus 2 randomly-ordered experimental trials separated by a 7-day washout period. Participants received either 6 (body mass < 91 kg) or 8 (body mass ≥ 91 kg) tablets of the treatment (TR; 625 mg of adenylpyrophosphoric acid and calcium pyruvate, 350.8 mg of cordyceps sinensis extract and yohimbine hydrochloride) or placebo (PL; 980 mg of microcrystalline cellulose) 1 hour prior to the following tests: countermovement vertical jump (CVJ), forearm flexion repetitions to exhaustion, isometric maximal voluntary contractions (MVCs) of the leg extensors, and a 50-repetition maximal concentric isokinetic leg extension endurance test.

**Results:**

There were no differences between the TR and PL trials for CVJ height (*P *> 0.05), isometric MVC peak torque (*P *> 0.05), maximal concentric isokinetic peak torque (*P *> 0.05), percent decline during the leg extension endurance tests (*P *> 0.05), or repetitions to exhaustion during the forearm flexion endurance tests (*P *> 0.05).

**Conclusion:**

These findings indicated no improvements in the measured variables as a result of ingesting this nutritional supplement. Future studies should examine whether chronic supplementation or a loading period is necessary to observe any ergogenic effects of this supplement.

## Background

Nutritional supplements are commonly used by recreational and competitive athletes as ergogenic aids to improve their physique and performance capabilities. For example, creatine is a widely used nutritional supplement that has been proven in multiple studies to increase skeletal muscle phosphocreatine and free creatine concentrations, which may enhance the ability to sustain high adenosine triphosphate (ATP) turnover rates during strenuous exercise [1]. As a result creatine supplementation may delay neuromuscular fatigue [2], improve muscle strength and power output [3], and increase muscle size [4]. The various purported benefits of nutritional supplements, such as creatine [5], have subsequently led the industry to explore the potential for alternative nutritional supplements that may yield similar benefits (i.e., the next 'creatine'). Among such nutritional supplements are those that are intended to directly increase ATP concentrations.

ATP is a purine nucleotide found in every human cell. The most important function of ATP is to transfer energy [6]. Subsequently, ATP is regarded as the smallest form of energy currency in living organisms [7]. When ATP is hydrolyzed to adenosine diphosphate (ADP) and inorganic phosphate (P_i_), the terminal phosphate bond is broken and energy is liberated to drive biological processes [7]. For human performance, the energy liberated by ATP hydrolysis for muscle contraction is of paramount importance [8]. Skeletal muscle concentrations of ATP are relatively limited, thus ATP must be continually resynthesized [9]. Thus, in theory, any nutritional supplementation that could increase ATP concentrations could also enhance performance during high-intensity exercise [8].

Kichenin et al. [10] examined the effects of oral ATP supplementation (5 mg/kg of [^14^C]ATP {1.85–2.29 GBq/mmol} and [^14^C]adenosine {1.85–2.29 GBq/mmol}) in rats. After 30 days of supplementation, there was an increase in exportation of ATP from the gut and improved adenosine uptake, ATP synthesis, and ATP transfer by red cells [10]. Therefore, these findings [10] suggested that it is possible to observe a positive biological response from chronic oral ATP supplementation in rats. In humans, Jordan et al. [8] administered 2 doses of an oral ATP supplement (high dose = 225 mg; low dose = 150 mg) and reported no differences in whole blood concentrations of ATP immediately after the supplementation or after 14 days of supplementation. In addition, oral ATP supplementation did not alter bench press strength or endurance, and there were no changes in peak power, average power, or total work during repeated 30-s Wingate tests [8]. There was, however, an increase in bench press training volume observed after 14 days of oral ATP supplementation, which provided tentative evidence that the oral ATP supplementation may be efficacious. Overall, the findings of Jordan et al. [8] suggested that dosages of 225 mg or less of oral ATP supplementation may have been too small to demonstrate any substantial performance enhancement in humans. Besides Jordan et al. [8], we are not aware of any other studies that have investigated the effects of orally-administered ATP supplements in humans. Therefore, the purpose of the present study was to examine the acute effects of a commercially available nutritional supplementation intended to improve ATP concentrations on vertical jump height, isometric strength of the leg extensors, leg extension endurance, and forearm flexion endurance.

## Methods

### Participants

Twenty-four healthy men (mean age ± SD = 23 ± 4 yrs, stature = 181 ± 7 cm, and body mass = 82 ± 12 kg) volunteered for this investigation. None of the participants reported any current or ongoing neuromuscular diseases or musculoskeletal problems specific to the ankle, knee, or hip joints. Each participant completed a pre-exercise health and exercise status questionnaire and signed a written informed consent document. Twenty-one of 24 participants reported engaging in 1–7 hours of aerobic exercise, 12 of 24 reported 1–8 hours of resistance exercise, and 18 of 24 reported 2–7 hours of recreational sports per week, however, none of the participants were competitive athletes.

### Supplementation protocol

Two experimental supplements were used: a placebo (PL: 980 mg of microcrystalline cellulose) and a commercially available treatment (TR; 625 mg of AdenylPyro-G™ formula [adenylpyrophosphoric acid and calcium pyruvate], 350.8 mg of Cordy-cAMP™ formula [cordyceps sinensis extract and yohimbine hydrochloride]) (5-TETRA, Epic Nutrition, Jupiter, FL). Supplements were administered in uniform containers and identifiable only by alphabetic code to both the investigators and the subjects. In accordance with the label recommendations, experimental trials 1 and 2 were performed 1 hour after the consumption of either 6 (body mass < 91 kg) or 8 (body mass ≥ 91 kg) tablets of the TR or PL. One week after each experimental visit, each subject was asked if they have had any adverse events since the previous visit. The experimental supplements used during this investigation were donated.

### Research design

This study was conducted with a double-blinded, placebo-controlled, crossover design. All participants completed 2 randomly-ordered experimental trials. The trials were separated by a 7-day washout period and were performed at the same time of day (± 2 hours). Seven days prior to the first experimental trial, each participant visited the lab for a familiarization trial to practice the vertical jumps, isometric and isokinetic leg extension muscle actions, and the dynamic constant external resistance (DCER) forearm flexion muscle actions. Each participant was instructed to arrive for the experimental trials after a 4-hour fast so that the TR and PL tablets were administered with water on an empty stomach in the laboratory under the supervision of the investigators. Sixty minutes after ingestion of either the TR or PL, the experimental trial began with a 5-minute warm-up on a stationary cycle ergometer (Monark 818E, Sweden) with a workload of 50 watts and cadence of 60–70 rpm. Following the warm-up, subjects immediately completed the following tests: vertical jump, DCER forearm flexion (i.e., biceps curls) repetitions to exhaustion, isometric maximal voluntary contractions (MVCs) of the leg extensors, and a 50-repetition isokinetic leg extension endurance test. All 4 assessments were performed in the same fashion for both trials with a 5-minute rest between each test.

### Vertical jump

Four maximal countermovement vertical jump (CVJ) trials where performed on a Just Jump™ mat (Probotics, Inc., Huntsville, AL) with a 1 minute rest period between trials. The Just Jump™ mat calculated CVJ height (cm) based on the flight time, which was the time that elapsed from the instant the feet left the mat until landing. To complete the CVJ trials, the participants stood on the mat with the feet shoulder width apart and the hands on the hips. A rapid descending quarter-squat countermovement was allowed prior to the ascending launch, however, no step was taken. The participants launched with both feet at the same time and landed in the same position. Two separate one-way ANOVAs were used to examine the systematic variation among the 4 CVJ trials for the TR and PL sessions. CVJ attempts 3 and 4 were greater than (*P *≤ 0.05) attempts 1 and 2, however, there were no difference (P > 0.05) between attempts 3 and 4. Therefore, the mean of CVJ trials 3 and 4 was used as the representative CVJ score.

### Isometric strength

Isometric torque for the right leg extensor muscles was measured using a Biodex System 3 isokinetic dynamometer (Biodex Medical Systems, Inc., Shirely, NY). The participants were seated with restraining straps over the pelvis, trunk, and contralateral thigh, and the lateral condyle of the femur was aligned with the input axis of the dynamometer in accordance with the Biodex User's Guide (Biodex Pro Manual, Applications/Operations. Biodex Medical Systems, Inc., Shirley, NY. 1998). All isometric torque assessments were performed at a leg flexion angle of 60° below the horizontal plane. Submaximal warm-up trials preceded two 4-s isometric MVCs of the right leg extensors. Participants were asked to produce as much force as possible for 4-s, and strong verbal encouragement was provided. Two minutes of rest was allowed between each MVC trial. The torque signal from the dynamometer was sampled at 1 KHz with an analog-to-digital converter (DAQcard™-6036E, National Instruments, Austin, TX) and custom software (LabVIEW Professional version 7.1, National Instruments, Austin, TX) and stored on a personal computer for off-line analysis. The torque signal was low-pass filtered (zero-phase 4^th^-order Butterworth filter) with a 10 Hz cutoff, and peak MVC torque (PT) was calculated as the highest average torque value that occurred during any 0.5-s duration within the 4-s MVC plateau. Two separate dependent samples *t *tests were used to examine whether there was any systematic variability between MVC trials 1 and 2 during the TR and PL sessions. There were no differences (P > 0.05) between the PT values for MVC trials 1 and 2; therefore, both trials were averaged and the mean was used as the representative PT value.

### Leg extension endurance

Five minutes after the isometric MVC trials, each participant completed the leg extension endurance test on the Biodex System 3 isokinetic dynamometer based on the procedures described by Housh, Housh, and deVries [11]. Three to five submaximal trials preceded 50 consecutive maximal concentric isokinetic leg extension muscle actions performed at 180°s^-1 ^with the right leg. The active range of motion was standardized from 90° to 180° of knee flexion and extension, respectively, for each participant. As with the isometric MVCs, the torque signal was sampled at 1 KHz and low-pass filtered (zero-phase 4^th^-order Butterworth filter) with a 10 Hz cutoff. PT was determined for each of the 50 repetitions during the extension muscle actions as the highest 10-ms average torque value that occurred during each torque curve. Not all subjects were able to complete all 50 repetitions; however, all subjects did complete at least 48 repetitions. Therefore, the first 48 repetitions were analyzed. The *initial *PT (IPT) was calculated as the average of the 3 highest PT values that occurred during the first 10 repetitions, whereas the *final *PT (FPT) represented the average of the 3 lowest PT values that occurred during the final 10 repetitions. Percent decline was calculated with the following equation [11]:

$$PercentDecline=\frac{IPT-FPT}{IPT}\times 100$$

### Forearm flexion endurance

During the familiarization trial, maximal bilateral strength of the forearm flexors was assessed with a standard one repetition maximum (1RM) procedure [12] in order to establish the same relative submaximal load for each subject for the TR and PL endurance tests. Each participant performed a light warm-up of 5 – 10 repetitions at approximately 50% of the estimated 1RM load. Following a 1-min rest, the load was increased for a second warm-up set of 3 – 5 repetitions. The load was then increased for the first 1RM attempt. If successful, 3 – 5 minutes was allowed before the next attempt with a load increase of 2.3 – 4.5 kg. This process continued until a failed attempt occurred, and the last successful lift was recorded as the representative 1RM score. The 1RM was determined within 5 attempts.

Using the 1RM value, repetitions of a bilateral forearm flexion exercise were performed until exhaustion with a load of 70% of the 1RM on an externally-loaded preacher curl machine (TDS™ Preacher Curl Machine, New York Barebell, Elmira, NY). The total number of repetitions performed throughout the full range of motion was recorded as the representative score. During the forearm flexion muscle actions, the participants were required to remain seated without leaning over the arm support. The participants were also required to keep their arms flat against the arm support while the lateral epicondyles of ulna remained aligned with the axis of rotation of the preacher curl machine. During the TR and PL trials, each participant performed a warm-up at 50% of the 1RM for 5–8 repetitions 2 minutes prior to the endurance test.

### Statistical analysis

Five separate paired-samples *t *tests were used to compare the CVJ height, isometric PT of the leg extensors, percent decline during the leg extension endurance test, and the repetitions to exhaustion during the forearm flexion endurance test scores for the TR vs. PL trials. A two-way repeated measures ANOVA (trial [TR vs. PL] × repetition [IPT vs. FPT]) was used to analyze the PT values during the leg extension endurance test. When appropriate, follow-up analyses included additional lower-order ANOVAs and paired samples *t *tests. SPSS software (SPSS, Inc., version 12.0, Chicago, IL) was used for all statistical analyses. The alpha level was set at *P *≤ 0.05.

## Results

There were no significant differences between the TR and PL trials for CVJ height (*P *> 0.05), isometric peak torque of the leg extensors (*P *> 0.05), percent decline during the leg extension endurance tests (*P *> 0.05), or repetitions to exhaustion during the forearm flexion endurance tests (*P *> 0.05) (Table 1). For IPT and FPT, there was no two-way interaction (trial × repetition, *P *> 0.05) or main effect for trial (*P *> 0.05); however, there was a decrease (*P *≤ 0.05) in the marginal means for peak torque (collapsed across trial) from IPT to FPT during the leg extension endurance test.

**Table 1:** Mean (SEM) values for tests following the PL and TR

**Test**	**PL**	**TR**
Countermovement Vertical Jump Height (cm)	51.4 (1.6)	51.6 (1.3)
Isometric Leg Extensor Strength (Nm)	186.8 (10.0)	187.5 (9.6)
Leg Extensor Endurance Test (% decline)	64.9 (1.7)	64.6 (1.6)
Forearm Flexion Endurance Test (# of reps)	11.8 (0.4)	11.7 (0.4)

## Discussion

In the present study we found that the oral ATP supplement, which was intended to improve performance, did not improve vertical jump height, isometric strength of the leg extensors, leg extension endurance, or forearm flexion endurance. Jordan et al. [8] examined the effects of different doses (high dose = 225 mg; low dose = 150 mg) of an orally-administered ATP supplement on muscular strength, anaerobic power, and anaerobic capacity. The high dose of oral ATP taken 75 minutes prior to testing resulted in an increase in bench press strength, which the authors attributed to the presence of two outliers and explained the improvement as a "...spurious change in the observed treatment differences, rather than one attributable to oral adenosine 5'-triphosphate treatment" (p. 988). There were no other changes reported for repetitions to exhaustion at 70% of 1RM or total resistance training volume. Similarly, there were no observed changes for any of the strength measurements in the low-dose group. Oral ATP supplementation did not improve peak power output, total work, or average power output during the two 30-s Wingate tests for either the high- or low-dose groups. After 14 days of high-dose oral ATP supplementation, total lifting volume increased by 22%, however, neither muscular strength, anaerobic power, or anaerobic capacity were influenced by the chronic supplementation [8]. The results of the present study extended the findings of Jordan et al. [8] and suggested that a much higher acute dose (5.9 – 7.8 g) of an oral ATP supplement did not improve muscular strength, endurance, or power output as measured by maximal voluntary isometric and isokinetic leg extension torque production, percent decline during the leg extension endurance test, repetitions to exhaustion for the biceps curl exercise, or CVJ height.

In previous studies, infusions of an ATP supplement have been reported to increase extracellular and intracellular ATP availability in rodent skeletal muscle by 116% and 50–70%, respectively [13,14]. However, Jordan et al. [8] administered an oral ATP supplement to humans and reported no differences in whole blood concentrations of ATP following acute or chronic supplementation. Since our findings suggested that an acute dose of this oral ATP supplement did not improve exercise performance (contrary to the label claim), it is possible that it did not increase ATP availability. Therefore, one hypothesis as to why the current oral ATP did not improve exercise performance is that the ingredients that were purported to increase ATP availability did not survive the process of digestion and absorption in the human gut [8]. A limitation of the present study was that we did not extract tissue or blood samples from these subjects, thus, we have no direct evidence to suggest that this supplement altered ATP concentrations. In contrast, the primary benefit of this applied study was that we tested (and ultimately refuted) the label claim that this oral ATP supplement improves performance. Since, at the time of this study, the oral ATP supplement we tested was available on the market, consumers may find our findings useful for making an informed purchase. To elucidate the potential for oral ATP supplementation to enhance performance, future studies should examine whether the acute or chronic ingestion of orally-administered ATP supplements increase extracellular and/or intracellular ATP concentrations, and subsequently, whether this translates into improved performance measures.

## Conclusion

In conclusion, there were no improvements in muscle strength, power output, or endurance as a result of ingesting 5.9 – 7.8 g of a nutritional supplement intended to increase ATP concentrations and availability. Theoretically, increases in ATP availability should improve human performance, however, lower doses of an oral ATP supplement have not been shown to improve whole blood ATP levels [8]. Although an acute dosage of this nutritional supplement showed no beneficial effects on performance, there were no detrimental effects either. In addition, no adverse events were reported during the course of this study, which suggested that acute doses may not be harmful. It is unclear whether the chronic ingestion of this supplement would result in any adverse effects. One difference between the present study and the methods of Jordan et al. [8] was that 60 minutes were allowed after ingestion prior to the performance measures, whereas Jordan et al. [8] allowed 75 minutes. Nevertheless, neither study demonstrated any compelling results to suggest that acute doses of orally-administered supplements that are marketed to improve ATP availability actually improve performance. Jordan et al. [8] did report an increase in total resistance training volume following a 14-day supplementation period. It is possible that a chronic loading period of 14 days or longer is necessary to demonstrate any meaningful ergogenic benefits. Therefore, future studies should examine whether a chronic loading period is necessary to observe any ergogenic effects of nutritional supplements that are marketed to improve ATP availability.

## Authors' contributions

TJH was the primary author of the manuscript.

EDR played an important role in data collection and manuscript preparation.

JRS played an important role in study design and manuscript preparation.

JTC was the senior author and played an important role in study design, data collection, data analysis and interpretation, and manuscript preparation.

All authors have read and approved the final manuscript.

## References

[B1] Volek JS, Kraemer WJ (1996). Creatine supplementaiton: Its effect on human muscular performance and body composition. J Strength Cond Res.

[B2] Stout J, Eckerson J, Ebersole K, Moore G, Perry S, Housh T, Bull A, Cramer J, Batheja A (2000). Effect of creatine loading on neuromuscular fatigue threshold. J Appl Physiol.

[B3] Earnest CP, Snell PG, Rodriguez R, Almada AL, Mitchell TL (1995). The effect of creatine monohydrate ingestion on anaerobic power indices, muscular strength and body composition. Acta Physiol Scand.

[B4] Volek JS, Duncan ND, Mazzetti SA, Staron RS, Putukian M, Gomez AL, Pearson DR, Fink WJ, Kraemer WJ (1999). Performance and muscle fiber adaptations to creatine supplementation and heavy resistance training. Med Sci Sports Exerc.

[B5] Stout JR, Antonio J, Kalman D (2007). Essentials of Creatine.

[B6] Brooks GA, Fahey TD, Baldwin KM, Barrosse E (2005). Exercise Physiology: Human Bioenergetics and Its Application.

[B7] McArdle WD, Katch FI, Katch VL, Darcy P (2001). Exercise Physiology: Energy, Nutrition, and Human Performance.

[B8] Jordan AN, Jurca R, Abraham EH, Salikhova A, Mann JK, Morss GM, Church TS, Lucia A, Earnest CP (2004). Effects of oral ATP supplementation on anaerobic power and muscular strength. Med Sci Sports Exerc.

[B9] Parkin JM, Carey MF, Zhao S, Febbraio MA (1999). Effect of ambient temperature on human skeletal muscle metabolism during fatiguing submaximal exercise. J Appl Physiol.

[B10] Kichenin K, Seman M (2000). Chronic oral administration of ATP modulates nucleoside transport and purine metabolism in rats. J Pharmacol Exp Ther.

[B11] Housh TJ, Housh DJ, Devries HA (2006). Applied Exercise & Sport Physiology.

[B12] Cramer JT, Coburn JW, Earle R, Baechle TR (2004). Fitness Testing Protocols and Norms. NSCA's Essentials of Personal Training.

[B13] Agteresch HJ, Dagnelie PC, Rietveld T, van den Berg JW, Danser AH, Wilson JH (2000). Pharmacokinetics of intravenous ATP in cancer patients. Eur J Clin Pharmacol.

[B14] Haskell CM, Wong M, Williams A, Lee LY (1996). Phase I trial of extracellular adenosine 5'-triphosphate in patients with advanced cancer. Med Pediatr Oncol.

